# Transcriptome analysis revealed differentially expressed genes in rice functionally associated with brown planthopper defense in near isogenic lines pyramiding *BPH14* and *BPH15*

**DOI:** 10.3389/fpls.2023.1250590

**Published:** 2023-08-08

**Authors:** Liang Hu, Dabing Yang, Hongbo Wang, Xueshu Du, Yanming Zhang, Liping Niu, Bingliang Wan, Mingyuan Xia, Huaxiong Qi, Tongmin Mou, Aiqing You, Jinbo Li

**Affiliations:** ^1^ Key Laboratory of Crop Molecular Breeding, Ministry of Agriculture and Rural Affairs, Hubei Key Laboratory of Food Crop Germplasm and Genetic Improvement, Food Crops Institute, Hubei Academy of Agricultural Sciences, Wuhan, China; ^2^ National Key Laboratory of Crop Genetic Improvement, Huazhong Agricultural University, Wuhan, China; ^3^ State Key Laboratory of Hybrid Rice, College of Life Sciences, Wuhan University, Wuhan, China; ^4^ Hubei Hongshan Laboratory, Wuhan, China

**Keywords:** rice, brown planthopper, RNA-sequencing, *BPH14/BPH15*, resistance

## Abstract

Although rice has many pests, brown planthopper (BPH) in particular is known to cause substantial damage. The pyramiding application of BPH-resistance genes *BPH14* and *BPH15* has proven effective in enhancing rice defense against BPH. However, the molecular mechanisms underlying *BPH14*/*BPH15*-conferred resistance remain unexplained. In this investigation, we analyzed the transcriptomes of near isogenic lines (NILs) containing either *BPH14* (B14), *BPH15* (B15), or *BPH14/BPH15* (B1415), as well as their recurrent parent (RP) ‘Wushansimiao’. In total, we detected 14,492 differentially expressed genes (DEGs) across 12 mRNA profiles of resistant NILs and RP at different feeding stages. In the transcriptomic analysis, 531 DEGs appeared to be common among the resistant NILs compared to RP before and after BPH feeding. These common DEGs were enriched in defense response, phosphorylation, and salt stress response. In addition, 258 DEGs shared only in resistant NILs were obtained among the different feeding stages, which were enriched in oxidative stress response, karrikin response, and chloroplast organization. Considering the expression patterns and relevant research reports associated with these DEGs, 21 were chosen as BPH resistance candidates. In rice protoplasts, the candidate DEG *OsPOX8.1* was confirmed to increase reactive oxygen species (ROS) accumulation by chemiluminescence measurement. Our results provide valuable information to further explore the defense mechanism of insect-resistant gene pyramiding lines and develop robust strategies for insect control.

## Introduction

Worldwide, more than 3.5 billion people utilize rice (*Oryza sativa* L.) as a dietary staple (Wing et al., 2018). Among all rice pests, one of the most damaging is the brown planthopper (*Nilaparvata lugens* Stål, BPH) (Du et al., 2020). As typical sap-sucking insects, BPHs gather in large numbers at the plant base and feed on phloem sap. This type of herbivory causes the drying, browning, wilting, and dwarfing of host plants. Extensive herbivory by BPH can ultimately lead to reduced or no yields, which seriously threatens food security (Cheng et al., 2013b). In addition, BPH can spread and induce various rice diseases, such as grassy dwarf disease and leaf dwarf disease (Jing et al., 2017). Breeding BPH resistant rice varieties is considered a practical, economical, and sustainable management strategy (Du et al., 2020).

In 1969, the International Rice Research Institute (IRRI) first discovered and mapped the *BPH1* BPH resistance gene, which paved the way for future studies of rice resistance to BPH. So far, 17 BPH resistance genes (*BPH37, BPH40, BPH30*, *BPH6*, *BPH32*, *BPH18*, *BPH21*, *BPH10*, *BPH7*, *BPH1*, *BPH9*, *BPH29*, *BPH3*, *BPH26*, *BPH2*, *BPH15*, and *BPH14*) have been successfully cloned in rice (Guo et al., 2022). Of these cloned genes, the majority represent coiled-coil nucleotide-binding site leucine-rich repeat (CC-NBS-LRR) proteins (e.g., *BPH14*), two encode lectin receptor-like kinases (LecRKs) (*BPH3* and *BPH15*), and the remainder encode other types of proteins (Guo et al., 2022). These BPH resistance proteins have diverse structures and functions, and the study of their varied molecular mechanisms can help us to better utilize them in precision breeding schemes.

The *BPH14* gene was the first to be cloned and encodes a nuclear/cytoplasmic CC-NBS-LRR protein which directly binds BPH-derived effector BISP to activate host plant resistance (Du et al., 2009; Guo et al., 2023). Through forming homologous complexes and interacting with transcription factors, BPH14 mediates BPH resistance by triggering the transcription of downstream defense genes (Hu et al., 2017). Meanwhile, *BPH15* encodes a plasma membrane LecRK which is suggested to serve as either a receptor or receptor-associated protein. As such, *BPH15* confers durable, broad-spectrum protection against BPH, as well as other pathogens, by perceiving either plant-derived damage-associated molecular patterns (DAMPs) or BPH-derived herbivore-associated molecular patterns (HAMPs). Furthermore, *BPH15* knock-down makes rice plants more susceptible to BPH and other pathogens (Cheng et al., 2013a).

Plants carrying only a single insect-resistance gene have the potential to become susceptible within a timeframe as short as a few years due to the adaptation of associated insect populations (Jena and Kim, 2010). One effective strategy to provide durable, broad-spectrum BPH protection in rice is the pyramiding of diverse resistance genes (Muduli et al., 2021). Marker-assisted pyramiding of rice with both *BPH14* and *BPH15* resulted in durable and enhanced resistance compared to rice varieties possessing only one of the two genes (Li et al., 2011; Hu et al., 2012; Jiang et al., 2018). In addition, varieties harboring two BPH resistance genes showed a more than 90% reduction in pest density in the field (Zheng et al., 2021). Using a genomics-based breeding approach, Wang et al. precisely incorporated *BPH14* and *BPH15* into recurrent parent (RP) ‘Wushansimiao’ rice to augment BPH resistance while leaving other agronomic traits unaffected (Wang et al., 2019). Unfortunately, the precise molecular mechanisms resulting in the enhanced BPH resistance of *BPH14*/*BPH15* pyramiding lines remain largely unknown.

In order to study these defense mechanisms, RNA sequencing (RNA-seq) has been successfully employed to characterize the rice transcriptome at different BPH feeding stages (Chen et al., 2022). For instance, the introgression line ‘B5’ contains five quantitative trait loci (QTL) and two major resistance genes (*BPH14* and *BPH15*) associated with resistance to BPH (Huang et al., 2001; Ren et al., 2004). Both cDNA macroarray and microarray analyses were performed to explore differential transcription between resistant cultivar ‘B5’ and susceptible cultivar ‘MH63’ under both BPH herbivory and insect-free conditions. Herbivory by BPH was found to affect a wide variety of gene functional categories, including pathogen-related proteins, oxidative stress, and signaling pathways, among others, suggesting that the adaptation of BPH-infested rice likely involves many pathways and processes (Zhang et al., 2004; Wang et al., 2008). In another experiment, high-throughput RNA-seq was used to discover nearly 3,000 BPH-responsive differentially expressed genes (DEGs) between a *BPH15* introgression line and recipient line. The identified DEGs were associated with a number of Gene Ontology (GO) terms, including hormone signaling, posttranslational protein modifications, transcription factors, pathogen-related genes, Ca^2+^ signaling, and MAPK cascades (Lv et al., 2014). A number of BPH-responsive miRNAs were identified by analyzing the miRNA profiles of a *BPH15* introgression line and susceptible recipient line, which were suggested to regulate several pathways contributing to both basal and BPH-specific defense (Wu et al., 2017). Furthermore, by combining microRNA and transcriptome analyses, 34 miRNAs associated with 42 target genes were identified as potential miRNA-mRNA pairs regulating *BPH6*-mediated resistance, implying the importance of miRNA-mRNA modules in regulating BPH defense (Tan et al., 2020).

Although *BPH14* and *BPH15* have been pyramided into rice varieties to confer durable and stable BPH resistance (Li et al., 2011; Hu et al., 2012; Wang et al., 2016; Wang et al., 2019; Yang et al., 2022), the precise molecular mechanism underlying the BPH resistance of *BPH14*/*BPH15* pyramiding lines remain largely unknown. Here, we analyzed the transcriptomes of near isogenic lines (NILs) containing either *BPH14*, *BPH15*, or both *BPH14*/*BPH15* genes, as well as their RP, before and after BPH infestation. Upon comparison and integration of these four datasets, a total of 21 DEGs were identified as candidates to functionally associate with rice defense against BPH. The data presented here help clarify the mechanism responsible for durable, broad-spectrum BPH resistance in gene pyramiding rice varieties.

## Materials and methods

### Experimental materials

The NILs containing either *BPH14* (B14), *BPH15* (B15), or both *BPH14*/*BPH15* (B1415) genes were developed using inbred *indica* rice variety ‘Wushansimiao’, as the RP (Wang et al., 2019). Seeds were planted in plastic cups (15 cm high by 9 cm wide) at a density of 15 plants per cup, and greenhouse-grown under a 10 h dark (26 ± 2°C)/14 h light (32 ± 2°C) cycle. The BPHs were maintained at Wuhan University, China, on ‘Taichung Native1’ (TN1; susceptible cultivar, IRRI Acc. No.00105) under environmental conditions identical to those of the rice plants.

### BPH resistance evaluation

BPH nymphs (third instar) were introduced at a rate of 8 BPH per seedling to four-leaf stage B14, B15, B1415, and RP seedlings. As described previously (Huang et al., 2001), seedlings were ascribed a resistance score during examination. The average damage severity score (0, 1, 3, 5, 7, or 9) was calculated for each plant after infestation.

### Honeydew excretion measurements

Pre-weighed parafilm sachets were used to confine starved (2 h) third instar BPH nymphs and fastened to the leaf sheathes of one-month-old B14, B15, B1415, and RP plants (Pathak et al., 1982). The sachets were removed and emptied of BPH insects after 2 d of active herbivory. All sachets were weighed post BPH removal, and the weight difference before and after 2 d of herbivory was recorded as the amount of honeydew excretion.

### Sample collection

Both BPH treatment and sample collection were accomplished according to the endpoint method (Wu et al., 2017). All treatments ended at the same time, despite beginning at different times. After 0, 3, 6, 12, 24, 48, and 72 h, four-leaf stage B14, B15, B1415, and RP seedlings were infested at a rate of 8 BPH nymphs (third instar) per seedling. Each experiment consisted of three biological replicates per treatment, with each replicate containing 15 seedlings. Leaf sheath samples were designated as either the non-infested group (0 h), early infestation group (3, 6, and 12 h), or late infestation group (24, 48, and 72 h). The experimental sample designations were as follows: B14_0, B14_early, and B14_late for the B14 lines; B15_0, B15_early, and B15_late for the B15 lines; B1415_0, B1415_early, B1415_late for the B1415 lines; and RP_0, RP_early, and RP_late for the RP lines. All samples were frozen with liquid N_2_ and stored at -80°C prior to analyses.

### RNA collection

Total RNA was collected from leaf sheathes with Trizol (Invitrogen). Quality was established with a Bioanalyzer 2200 (Aligent). All samples were stored at -80°C prior to analyses.

### cDNA library preparation

A TruSeq Stranded mRNA Library Prep Kit (Illumina) was utilized for preparation of the cDNA libraries, according to the standard protocol. Briefly, oligo (dT) magnetic beads were utilized to purify poly-A mRNA from 1 μg total RNA, which was then fragmented (200-600 bp) for 6 min with divalent cations (85°C). Both first- and second-strand cDNA synthesis were carried out using the cleaved RNA fragments. dUTP mix was utilized for second-strand cDNA synthesis, allowing for second strand separation. The cDNA fragments were then ligated with indexed adapters, A-tailed, and end-repaired. To remove the second-strand cDNA, the ligated cDNA was purified and subjected to uracil DNA glycosylase. The cDNA libraries were created by using PCR to enrich the purified first-strand cDNA. An Agilent 2200 was used for library quality control, and the libraries were sequenced using NovaSeq 6000 on a 150 bp paired-end run.

### RNA sequence mapping

Adapter sequences and low-quality reads were removed in order to acquire clean reads. Hisat2 was utilized to align the clean reads with the reference genome (IRGSP1.0, Ensembl) (Kim et al., 2015). Gene counts were acquired with HTseq. Gene expression was quantified according to the fragments per kilo base of exon per million fragments mapped (FPKM) (Anders et al., 2015).

### Differential gene expression analysis

DEGs were filtered using the DESeq2 algorithm (Love et al., 2014). Statistically significant DEGs were determined according to *P*-value (< 0.05), fold change (FC; log_2_FC > 1 or log_2_FC < -1), and FDR (< 0.05) (Benjamini et al., 2001). Here, DEGs are defined as transcripts exhibiting a P-value < 0.05 and at least a 2-fold change in FPKM (log_2_FC > 1 or log_2_FC < -1).

### Gene Ontology (GO) evaluation

GO evaluation was carried out to elucidate the biological importance of the identified DEGs (Ashburner et al., 2000), using GO annotations downloaded from the Gene Ontology (http://www.geneontology.org/), UniProt (http://www.uniprot.org/), and NCBI (http://www.ncbi.nlm.nih.gov/) databases. Statistically significant GO categories were determined with the Fisher’s exact test (*P*-value < 0.05).

### Kyoto Encyclopedia of Genes and Genomes (KEGG) pathway evaluation

KEGG pathway evaluation was carried out to determine the biological pathways associated with the identified DEGs according to the KEGG database. Statistically significant KEGG pathways were determined with the Fisher’s exact test (*P*-value < 0.05) (Draghici et al., 2007).

### Quantitative real-time PCR (qRT-PCR) assay

A PrimeScript RT Reagent Kit containing gDNA Eraser (RR047A, TaKaRa) was used to convert total RNA into first-strand cDNA. qRT-PCR was accomplished on a CFX96 real-time system (Bio-Rad) with SYBR Green Real-Time PCR Master Mix (QPK-201, Toyobo). All primers are listed in **Supplementary Table 1**. Gene expression was evaluated by relative quantification, with *TBP* as the endogenous reference (Livak and Schmittgen, 2001).

### Gene constructs and transformation

The *NB* domain of *BPH14* and the *OsPOX8.1* coding sequence were amplified from ‘B5’ and ‘Wushansimiao’ cDNAs, and then respectively cloned into the ZeBaTA-based pCXUN expression vector with a Myc tag at the c-terminus (Chen et al., 2009). All primers are listed in **Supplementary Table 1**. The aforementioned constructs were transiently transfected into 10-day old rice stem protoplasts as described previously (Chen et al., 2006).

### Protein collection and protein gel blot assay

Transfected protoplasts were extracted using a protein extraction buffer containing 5 mM MgCl_2_, 100 mM Tris-HCl (pH 7.5), 0.5% (w/v) Triton X-100, and 1 mM EDTA, with 1 mM PMSF and 2 mM DTT included just prior to the assay. Total soluble proteins were collected from rice protoplasts (5×10^6^ cells per sample) using 200 μL of extraction buffer. SDS-PAGE was carried out to separate 10 μL of the extract. The extract was diluted (1:1000) with dilution buffer (3% [w/v] BSA, 150 mM NaCl, 0.1% [w/v] Tween 20, 20 mM Tris-HCl [pH 7.4]) and used for anti-Myc antibody (M192-3, MBL) immunoblotting, and subsequently incubated with 5% (w/v) skim milk-diluted (1:10,000) secondary antibody conjugated to horseradish peroxidase (115-035-003, Jackson). Detection was carried out using Tanon high-sig ECL protein gel blotting substrate.

### Statistical analyses and reproducibility

All experiments consisted of three biological replicates, except where stated otherwise. Equivalent results were obtained using three independent biological experiments. Statistically significant differences were identified using Student’s t-tests at *P* value < 0.05.

### Reactive oxygen species (ROS) assay

**Figure 1:**
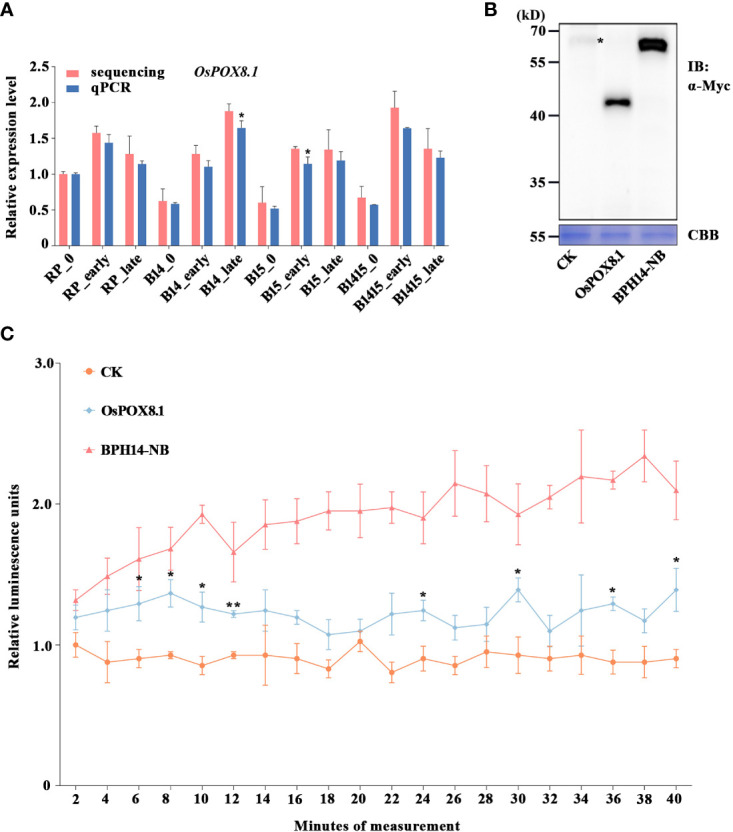
Verification of candidate DEG *OsPOX8.1* related to defense response. **(A)** qRT-PCR was used to verify the mRNA expression pattern of *OsPOX8.1* in the RP, B14, B15, and B1415 plants. The rice *TBP* gene was used as a reference control. Gene expression was quantified relative to the value obtained from non-infested RP samples. Data represent the means of three biologically independent experiments for gene expression ± SD. Data were subjected to Student’s t-test, and asterisks indicate significant differences between RNA-seq data and qRT-PCR data at the indicated group (*P < 0.05; **P < 0.01). **(B)** Protein immunoblotting of empty vector (CK)*, OsPOX8.1*, and NB domain of BPH14 (BPH14-NB) expressed in rice protoplasts. Asterisks indicate nonspecific signals. Coomassie brilliant blue (CBB) staining served as the loading control. Molecular masses (in kilodaltons) are indicated. **(C)** ROS generation in *OsPOX8.1-*overexpressing rice protoplast line. Relative luminescence units indicate relative amounts of ROS production in rice protoplasts at the indicated time points. Protoplast lines transformed with the empty vector and NB domain of BPH14 were used as negative control (CK) and positive control (BPH14-NB), respectively. Data represent the means of three technical replicates from one biological replicate ± SE. Three biologically independent experiments yielded similar results. Data were subjected to Student’s t-test, and ROS generation in the BPH14-NB protoplast line significantly differs from that in CK from the first time point onwards. Asterisks indicate significant differences between the OsPOX8.1 protoplast line and CK protoplast line at the indicated time point (*P < 0.05; **P < 0.01).

Evaluation of ROS production in rice protoplasts (as shown in **Figure 1C**) was carried out with a modified chemiluminescence method (Zhang et al., 2007). Briefly, the protoplasts were transfected for 16-22 h and then quantified and diluted to 1×10^5^ cells/200 μL with W5. To the diluted protoplasts was added 20 μM of the luminol derivative 8-amino-5-chloro-7-phenylpyrido [3,4-d] pyridazine-1,4 (2H,3H) dione (L-012) (Wako) and 20 μg/mL horseradish peroxidase (Sigma-Aldrich). Luminescence was captured using a SpectraMax iD5 multi-mode microplate reader (Molecular Devices).

## Results

### Performance of *BPH14*/*BPH15* pyramiding NILs against BPH

**Figure 2:**
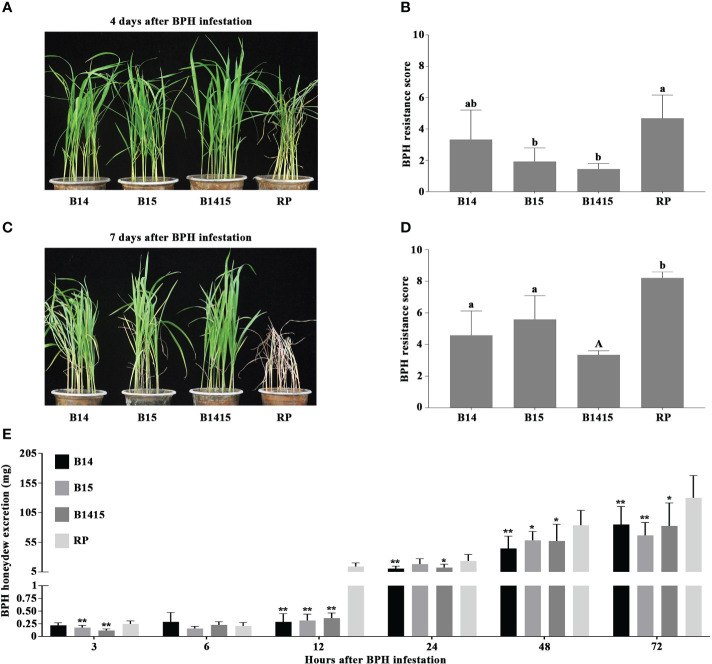
Evaluation of BPH resistance of the B14, B15, B1415, and RP plants. **(A)** BPH resistance phenotypes of the B14, B15, B1415, and RP plants after 4 days of BPH feeding. The image shows that the RP plants began to wither while the B14, B15, and B1415 plants showed no visible damage. RP: recurrent parent ‘Wushansimiao’ for NILs; B14, B15, and B1415: the NILs containing the *BPH14*, *BPH15*, and both *BPH14/BPH15* genes, respectively. **(B)** BPH resistance scores of the B14, B15, B1415, and RP plants after 4 days of BPH feeding. The resistance scores of B14, B15, B1415, and RP were 3.3, 2.0, 1.5, and 4.7, respectively. Lower scores correspond to higher levels of insect resistance. Data represent the means of three biologically independent experiments (with each experiment having 15 seedlings per rice line) ± SD. **(C)** BPH resistance phenotypes of the B14, B15, B1415, and RP plants after 7 days of BPH feeding. The image shows that the RP plants died while the B14, B15, and B1415 plants began to wither. **(D)** BPH resistance scores of the B14, B15, B1415, and RP plants after 7 days of BPH feeding. The resistance scores of B14, B15, B1415, and RP were 4.6, 5.6, 3.3, and 8.2, respectively. **(E)** Honeydew excretion of BPH insects on B14, B15, B1415, and RP plants after 2 days of feeding. Data represent the means of 10 replicates (with each replicate having one BPH insect per plant) ± SD. All data were subjected to Student’s t-test, different letters above the bars indicate significant differences between each line of plants **(B, D)** (uppercase letter *P* < 0.05; lowercase letter *P* < 0.01), and asterisks indicate significant differences between NIL and RP plants **(E)** (*P < 0.05; **P < 0.01).

In this study, four-leaf stage NILs containing either the *BPH14* (B14), *BPH15* (B15), or *BPH14/BPH15* genes (B1415), as well as their RP, were infested with BPH. RP plants began to wither after 4 d of BPH herbivory (average score of 4.7), and wilted completely after 7 d (average score of 8.2). However, the B14, B15 and B1415 plants showed no visible damage (average scores of 3.3, 2.0, and 1.5, respectively) and survived until the end of the experiment (average scores of 4.6, 5.6, and 3.3, respectively) (**Figures 2A–D**).

To investigate the antibiosis effects of the NIL and RP plants, we measured the quantity of BPH-secreted honeydew. Overall, BPH feeding on RP and NIL plants produced very little honeydew from 3 to 6 h after infestation. Interestingly, the most significant differences in honeydew production were observed at 12 h after infestation, with the amount of honeydew production remaining relatively constant on NIL plants (from a minimum of 0.16 mg at 6 h to a maximum of 0.36 mg at 12 h) and increasing sharply on RP plants (from a minimum of 0.21 mg at 6 h to a maximum of 14.4 mg at 12 h). After 12 h, honeydew production increased on both NIL and RP plants, and remained high from 24 to 72 h after infestation (**Figure 2E**).

To identify DEGs functionally associated with defense against BPH in NILs pyramiding the BPH14 and BPH15 genes, RNA was extracted from the leaf sheaths of B14, B15, B1415, and RP plants after infestation (0-72 h). Samples were grouped as non-infested (0 h), early feeding stage (3, 6 and 12 h), or late feeding stage (24, 48 and 72 h) for RNA-seq.

### Overview of the RNA-Seq results

Differences in BPH-responsive gene expression were analyzed using mRNA libraries. From 36 mRNA libraries, a total of 31,739,768 to 50,072,060 reads were sequenced. After removing low quality sequences, 82.38%-89.76%, 82.96%-90.53%, 81.69%-90.48%, and 87.83%-90.93% of the reads were mapped to 25,781,469-42,511,732 (RP), 32,708,227-43,047,757 (B14), 29,297,676-41,357,463 (B15), and 33,097,294-44,922,512 (B1415) rice genes, respectively (**Supplementary Table 2**).

**Figure 3:**
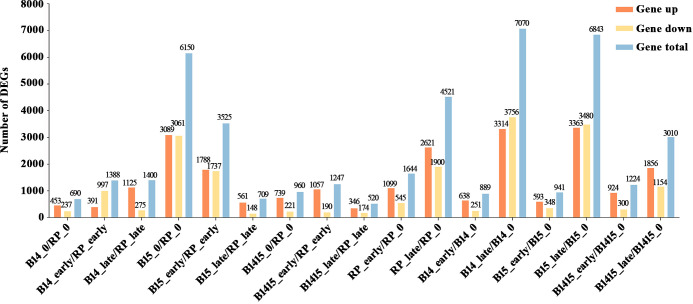
Contrast between up-regulated and down-regulated DEGs in all comparisons. “Gene up” represents the number of DEGs that were up-regulated in the compared group. “Gene down” represents the number of DEGs that were down-regulated in the compared group. “Gene total” represents the total number of DEGs in the compared group (log_2_FC > 1 or log_2_FC < -1; *P* < 0.05).

Subsequent to normalization, the average normalized reads from three independent biological replicates were selected for further studies. In total, 14,492 DEGs were identified among 17 comparisons, including nine comparisons among the different varieties (B14_0/RP_0, B14_early/RP_early, B14_late/RP_late, B15_0/RP_0, B15_early/RP_early, B15_late/RP_late, B1415_0/RP_0, B1415_early/RP_early, B1415_late/RP_late) and eight comparisons among the different feeding stages (RP_early/RP_0, RP_late/RP_0, B14_early/B14_0, B14_late/B14_0, B15_early/B15_0, B15_late/B15_0, B1415_early/B1415_0, B1415_late/B1415_0) (**Figure 3** and **Supplementary Table 3**).

A total of 690, 1,388, and 1,400 DEGs were identified in the B14_0/RP_0, B14_early/RP_early, and B14_late/RP_late comparisons, respectively; a total of 6,150, 3,235, and 709 DEGs were identified in the B15_0/RP_0, B15_early/RP_early, and B15_late/RP_late comparisons, respectively; and a total of 960, 1,247, and 520 DEGs were identified in the B1415_0/RP_0, B1415_early/RP_early, and B1415_late/RP_late comparisons, respectively (**Figure 3**). In addition, 6,165 DEGs were identified in RP plants (1,644 in RP_early/RP_0 and 4,521 in RP_late/RP_0), 7,959 DEGs were identified in B14 plants (889 in B14_early/B14_0 and 7,070 in B14_late/B14_0), 7,784 DEGs were identified in B15 plants (941 in B15_early/B15_0 and 6,843 in B15_late/B15_0), and 4,234 DEGs were identified in B1415 plants (1,224 in B1415_early/B1415_0 and 3,010 in B1415_late/B1415_0). These results illustrate that the DEGs were responsive to BPH feeding, with a higher response in B14 and B15 plants compared to RP, and a lower response in B1415 plants than in RP plants (**Figure 3**). These results suggest the presence of different modes of regulation at the early and late herbivory stages among the different rice plants.

### Reference gene selection and validation of DEGs

**Figure 4:**
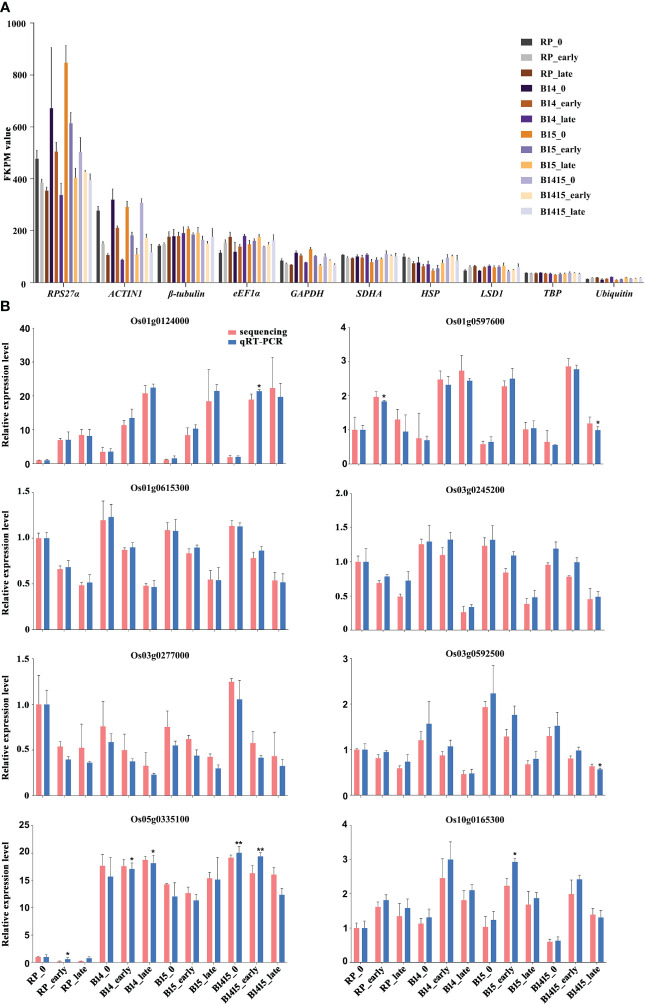
Expression profiles of mRNAs. **(A)** FKPM values of *RPS27α*, *ACTIN1*, *β-tubulin*, *eEF1α*, *GAPDH*, *SDHA*, *HSP*, *LSD1*, *TBP*, and *Ubiquitin* from RNA-seq data. **(B)** qRT-PCR was used to verify mRNA expression patterns in the RP, B14, B15, and B1415 plants. The rice *TBP* gene was used as a reference control. Gene expression was quantified relative to the value obtained from non-infested RP samples. Data represent the means of three biologically independent experiments for gene expression ± SD. All data were subjected to Student’s t-test, and asterisks indicate significant differences between RNA-seq data and qRT-PCR data for the indicated group (*P < 0.05; **P < 0.01).

During interactions between host plants and herbivores, reference gene expression is often suppressed (Hu et al., 2011). Normalization candidates were chosen after identifying which of the following common rice reference genes were the most stably-expressed: *RPS27α* (Os01g0328400), *ACTIN1* (Os03g0718100), *β-tubulin* (Os03g0780600), *eEF1α* (Os03g0177500), *GAPDH* (Os02g0601300), *SDHA* (Os07g0134800), *HSP* (Os03g0426900), LSD1 (Os12g0611000), *TBP* (Os03g0657000), and *Ubiquitin* (Os03g0131300). Each was evaluated using FPKM values extracted from the RNA-seq data. Overall, both *RPS27α* and *ACTIN1* expressions were significantly reduced after BPH herbivory in all groups. Compared with other candidates, *TBP* exhibited the most stable and appropriate expression level and was chosen as the endogenous reference gene for qRT-PCR validation assays (**Figure 4A**). The expression levels of eight DEGs were determined by qRT-PCR utilizing gene-specific primers (**Supplementary Table 1**) for RNA-seq verification, and we found that the data were in agreement (**Figure 4B**).

### Identification of BPH resistance DEGs among the different varieties

**Figure 5:**
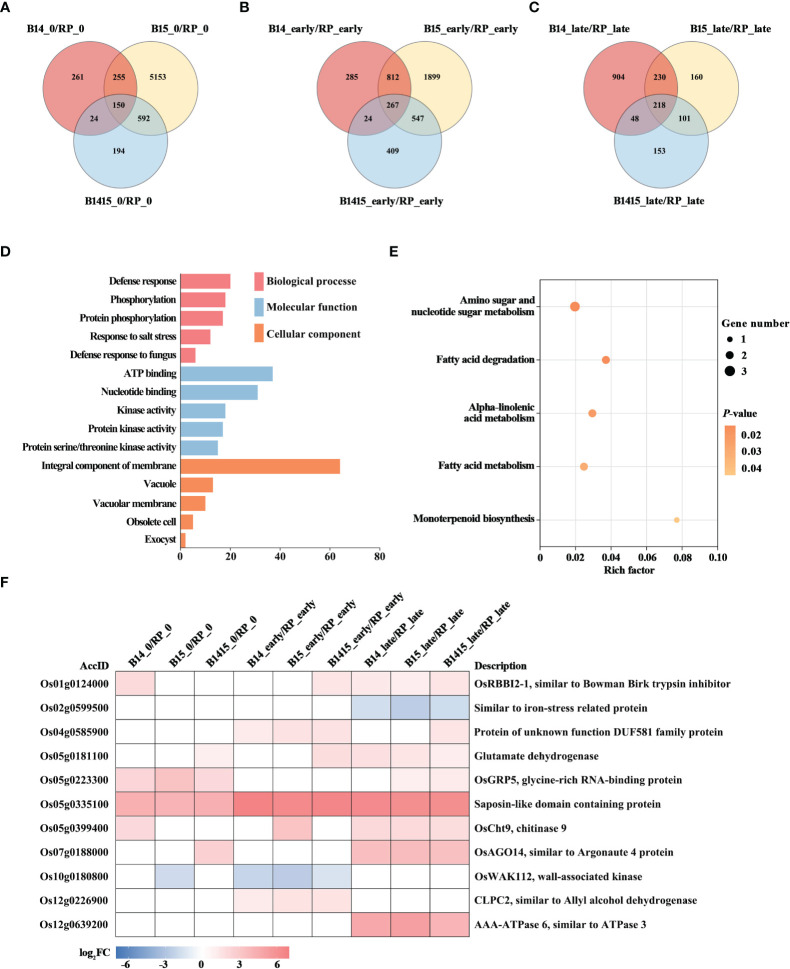
Analysis of DEGs related to BPH resistance among the different varieties. **(A-C)** Venn diagrams of the unique and shared DEGs among the different varieties. Venn diagram of the number of DEGs of the resistant NILs compared to RP at the non-infested stage **(A)**, early feeding stage **(B)**, and late feeding stage **(C)**. **(D)** Gene ontology (GO) analysis. Biological processes, molecular functions, and cellular components of the 531 common DEGs among the resistant NILs compared to RP before and after BPH feeding (*P* < 0.05). The x- and y-axes indicate the number of genes in a category and the names of the clusters, respectively. **(E)** Kyoto encyclopedia of genes and genomes (KEGG) analysis. KEGG pathway enrichment analysis of the 531 common DEGs among the resistant NILs compared to RP before and after BPH feeding (*P* < 0.05). The x- and y-axes indicate the rich factor of each pathway and the pathway name, respectively. The bubble size indicates the number of genes. The color bar indicates the *P*-value. **(F)** Hierarchical clustering analysis of 11 potential candidate DEGs related to BPH resistance among the different varieties. The color bar represents fold-change values shown in the log_2_ scale based on FPKM values.

To discover BPH resistance-associated genes, DEGs appearing in the comparisons of the resistant NIL vs. RP plants were analyzed by Venn diagrams, respectively (**Figures 5A–C**). We identified 150 overlapping DEGs in the B14_0/RP_0, B15_0/RP_0, and B1415_0/RP_0 comparisons (**Figure 5A**); 267 overlapping DEGs in the B14_early/RP_early, B15_early/RP_early, and B1415_early/RP_early comparisons (**Figure 5B**); and 218 overlapping DEGs in the B14_late/RP_late, B15_late/RP_late, and B1415_late/RP_late comparisons (**Figure 5C**). By combining these overlapping results, we obtained 531 DEGs common to B14, B15, and B1415 plants before and after BPH feeding, which may be involved in BPH resistance (**Supplementary Table 4**).

To functionally categorize these 531 DEGs, we analyzed their associated GO terms and KEGG pathways. The DEGs were mainly enriched in the defense response, phosphorylation, and salt stress response GO biological processes; in the ATP binding, nucleotide binding, and kinase activity GO molecular functions; and the integral component of membrane, vacuole, and vacuolar membrane GO cellular components (**Figure 5D**). For KEGG analysis, the BPH-responsive DEGs were found to be primarily enriched in alpha-linolenic acid metabolism, amino sugar and nucleotide sugar metabolism, fatty acid metabolism, fatty acid degradation, and monoterpenoid biosynthesis. (**Figure 5E**).

Finally, we comprehensively evaluated both the expression patterns of and the relevant research reports pertaining to the identified DEGs, and ultimately landed on 11 BPH resistance-related genes. Among these, nine were significantly up-regulated in the resistant NIL plants compared with RP plants before and after BPH herbivory, while two (Os02g0599500 and Os10g0180800) were down-regulated in the resistant NIL plants compared with RP plants before and after BPH herbivory (**Figure 5F**).

### Identification of BPH resistance DEGs among the different feeding stages

**Figure 6:**
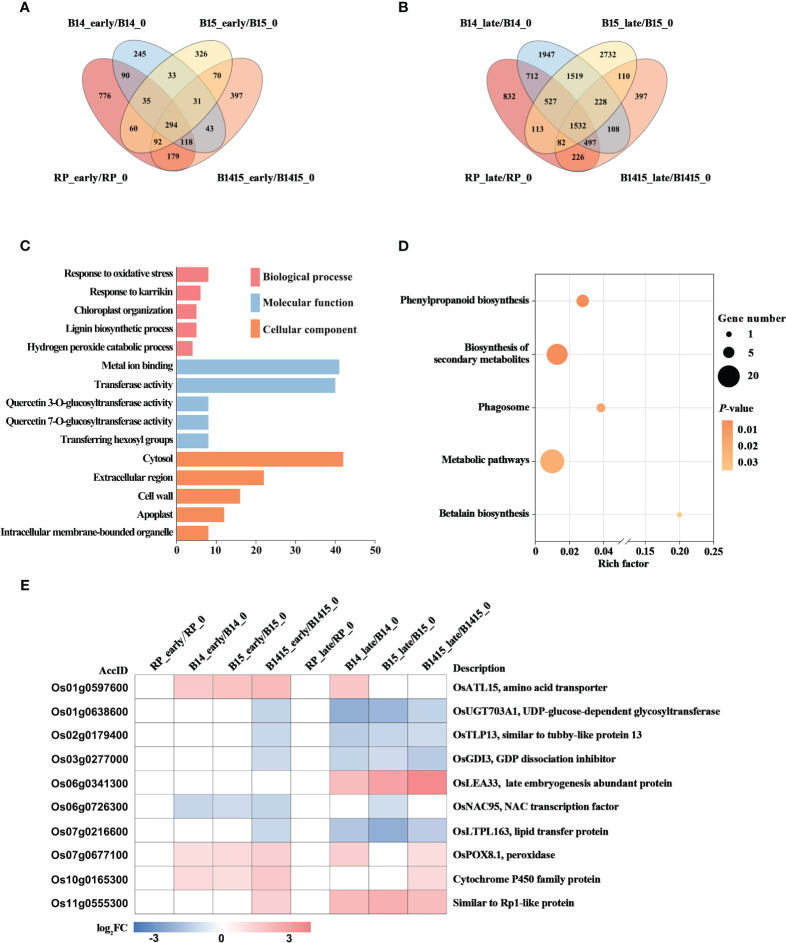
Analysis of DEGs related to BPH resistance among the different feeding stages. **(A, B)** Venn diagrams of the unique and shared DEGs among the different feeding stages. Venn diagram of the number of DEGs in early feeding stage **(A)** and late feeding stage **(B)** of the resistant NILs and RP compared to themselves at the non-infested stage. **(C)** Gene ontology (GO) analysis. Biological processes, molecular functions, and cellular components of the 258 DEGs which were shared only in resistant NILs obtained among the different feeding stages (*P* < 0.05). The x- and y-axes indicate the number of genes in a category and the names of the clusters, respectively. **(D)** Kyoto encyclopedia of genes and genomes (KEGG) analysis. KEGG pathway enrichment analysis of the 258 DEGs which were shared only in resistant NILs at the early or late stages of BPH feeding (*P* < 0.05). The x- and y-axes indicate the rich factor in each pathway and the pathway name, respectively. The bubble size indicates the number of DEGs. The color bar indicates the *P*-value. **(E)** Hierarchical clustering analysis of ten potential candidate DEGs related to BPH resistance among the different feeding stages. The color bar represents fold-change values shown in the log_2_ scale based on FPKM values.

The DEGs of both resistant NIL and RP plants at the early and late stages of herbivory were compared with those at the non-infested stage using Venn diagrams (**Figures 6A, B**). A total of 31 DEGs were specifically expressed in B14_early/B14_0, B15_early/B15_0, and B1415_early/B1415_0, while a total of 228 DEGs were specifically expressed in B14_late/B14_0, B15_late/B15_0, and B1415_late/B1415_0 (**Figures 6A, B**). The common DEGs were further pooled, and 258 DEGs were found to be shared only in resistant NILs during the early or late stages of BPH feeding (**Supplementary Table 5**).

To functionally categorize these 258 DEGs, we analyzed their associated GO and KEGG pathways. The DEGs were mainly enriched in the response to oxidative stress, chloroplast organization, and response to karrikin GO biological processes; the metal ion binding, transferase activity, and glucosyltransferase activity GO molecular functions; and the cytosol, cell wall, and extracellular region GO cellular components (**Figure 6C**). For KEGG analysis, the BPH-responsive DEGs were found to be primarily enriched in betalain biosynthesis, biosynthesis of secondary metabolites, metabolic pathways, phagosome, and phenylpropanoid biosynthesis (**Figure 6D**).

Finally, we comprehensively evaluated both the expression patterns of and the relevant research reports pertaining to the identified DEGs, and ultimately landed on 10 BPH resistance-related genes. Most of these candidates, excluding Os06g0341300, were rapidly up- or down-regulated during the early BPH feeding stage, specifically in resistant NIL plants, with significant differences remaining during the late herbivory stages (**Figure 6E**).

### Verification of candidate DEGs related to BPH resistance

Defense against BPH and other pathogens often involves the generation of ROS (Hu et al., 2017). Specifically, the rapid accumulation of ROS serves as a signal that coordinates an astonishing diversity of defense processes, while also being directly toxic to intruders (Gechev et al., 2006). The *OsPOX8.1* gene, encoding a class III peroxidase, is highly up-regulated in response to blast and bacterial blight, where it is involved in the generation of ROS (Yin et al., 2000; Xiao et al., 2015). Here, the BPH-responsive candidate gene *OsPOX8.1* was found to belong to the GO category “response to oxidative stress” (**Figure 6C**). Through qRT-PCR validation, we found that *OsPOX8.1* was significantly up-regulated by BPH herbivory only in B14, B15, and B1415 plants, and the degree of up-regulation was higher in B1415 plants than in B14 or B15 plants. Consistent with the RNA-seq results, *OsPOX8.1* expression was responsive from the early through the late feeding stages (**Figure 1A**).

To verify whether *OsPOX8.1* regulates ROS levels, rice protoplasts were first transformed with *OsPOX8.1*. An empty vector construct (control, CK) and an auto-activated construct of the NB domain of BPH14 (BPH14-NB) were utilized as negative and positive controls, respectively (Hu et al., 2017). According to the immunoblotting experiments, each of the transformed proteins exhibited expected expression patterns (**Figure 1B**). ROS production in the protoplast lines was measured histochemically using the chemiluminescence method. The protoplasts transformed with *OsPOX8.1* exhibited ROS accumulation, which was significantly stronger than that of protoplasts transformed with CK, but weaker than that of the protoplasts transformed with BPH14-NB (**Figure 1C**). These results indicated that *OsPOX8.1* enhanced ROS production in rice protoplasts.

## Discussion

Pyramiding lines containing both *BPH14* and *BPH15* exhibit more durable and effective protection than lines containing only *BPH14* or *BPH15* (Li et al., 2011; Hu et al., 2012; Jiang et al., 2018). However, the molecular mechanisms of BPH resistance underlying *BPH14*/*BPH15* pyramiding lines are poorly understood. This study is the first to perform an RNA-seq analysis of NILs containing either *BPH14* or *BPH15*, or both, as well as their RP. The data presented here aid our understanding of the regulatory mechanisms of BPH resistance gene pyramiding lines upon BPH attack.

Consistent with the previous study (Wang et al., 2019), performance and evaluation of *BPH14*/*BPH15* pyramiding NILs against BPH showed that pyramiding *BPH14* and *BPH15* in ‘Wushansimiao’ resulted in significantly enhanced resistance to BPH, with the B1415 plants exhibiting much stronger BPH resistance than the B14 or B15 plants (**Figure 2**). In addition, there were significant differences in honeydew production on the resistant NIL and RP plants 12 h after infestation (**Figure 2E**), these results suggested that stronger resistance factors (e.g. callose deposits on sieve plates) might exist to prevent the phloem sap ingestion by BPH from resistant NIL plants than from RP plants (Hao et al., 2008). Therefore, the RNA samples from the NIL and RP plants were categorized as either early feeding stage (before 12 h), late feeding stage (after 12 h), or non-infested.

By comparing mRNA expression between the B14, B15, B1415, and RP plants before and after BPH infestation, a total of 14,492 DEGs were identified among 17 comparisons (**Figure 3**). Although a comparison of the RNA-seq results between B1415 and RP plants was sufficient to identify BPH resistance-associated DEGs, studying the RNA-seq results of B14 and B15 plants may provide more details about the mechanism of rice resistance to BPH, and may also more accurately and reliably identify DEGs related to BPH resistance. There were fewer DEGs detected in the B1415_early/RP_early and B1415_late/RP_late comparisons than in the B14_early/RP_early and B14_late/RP_late comparisons or the B15_early/RP_early and B15_late/RP_late comparisons during the early and late feeding stages. These results indicate that the B1415 plants experienced less damage and had a relatively normal physiological status compared to the other plants due to their strong BPH resistance (**Figure 3**). Meanwhile, the B1415 plants had more up-regulated than down-regulated DEGs, implying that the expression of BPH resistance-related genes might be up-regulated in B1415 plants (**Figure 3**).

The selection of an appropriate reference gene which exhibits minimal changes in expression during a particular experiment is critical to the accuracy of qRT-PCR analyses. Various housekeeping genes show a certain degree of variability during plant-pathogen and plant-herbivore interactions (Hu et al., 2011). The expressions of some novel candidate reference genes were modified due to metabolic alterations and organ-specific gene expression reprogramming in response to invasion (Mascia et al., 2010). For instance, the conventional reference gene *ACTIN1* exhibits greater dynamic changes in infected plants due to its involvement in the transport of defense-related compounds (Henty-Ridilla et al., 2014). We compared the stability of ten novel reference gene candidates: *RPS27α, ACTIN1*, *β-tubulin*, *eEF1α*, *GAPDH*, *SDHA*, *HSP*, *LSD1*, *TBP*, and *Ubiquitin*. Upon comparison of the FPKM values extracted from the RNA-seq data, identical rankings were observed for the most stable reference gene *TBP*, which is in accordance with prior reports (Hu et al., 2011; Guo et al., 2018; Tan et al., 2020). In contrast, *RPS27α* and *ACTIN1* were ranked among the least stable, suggesting that these genes experience highly variable expression during BPH infestation.

A total of 531 DEGs appeared in B14, B15, and B1415 plants, compared to RP plants, before and after BPH infestation. In addition, a greater number of overlapping DEGs were identified in comparisons of different varieties during BPH feeding (267 and 218 overlapping DEGs, as shown in **Figures 5B, C**, respectively) than before BPH feeding (150 overlapping DEGs, as shown in **Figure 5A**), suggesting that many DEGs were activated to defend against BPH infestation. These DEGs were most enriched in defense response (GO), which is consistent with the above conclusion (**Figure 5D**). In addition, KEGG pathway analysis suggested that the responses of resistant NILs against BPH were compensatory or tolerance-enhancing in nature. Specifically, these DEGs were found to be related to alpha-linolenic acid metabolism, amino sugar and nucleotide sugar metabolism, fatty acid metabolism, fatty acid degradation, and monoterpenoid biosynthesis (**Figure 5E**). Based on the expression patterns of, and relevant references pertaining to, the above DEGs, 11 genes were chosen as potential BPH resistance candidates (**Figure 5F**). The Bowman-Birk trypsin inhibitor plays a role in the plant biotic stress response by inhibiting trypsin activity (Pang et al., 2013). Iron stress can activate the immune response, and plants may recognize pathogens by way of iron depletion (Herlihy et al., 2020). In *Arabidopsis*, *increased resistance to myzus persicae 1* (*IRM1*) (encoding DUF581 domain-containing protein) overexpression confers aphid resistance (Chen et al., 2013). Furthermore, *glycine-rich RNA-binding protein* (*GRP*) gene knock-out *Arabidopsis* lines are less resistant to *Pseudomonas* (Fu et al., 2007). Egg production and embryonic development of *Meloidogyne incognita* is reduced by chitinase gene expression (Chan et al., 2010). In response to herbivory, Argonautes (AGOs) modulate several defense regulation nodes (Pradhan et al., 2017). In rice, the wall-associated kinases (WAKs) act as both negative and positive regulators of fungal defense (Delteil et al., 2016). Disease susceptibility, the hypersensitive response, and pathogen growth are activated by co-suppression of *CLPC1* and *CLPC2* (Ali et al., 2019). In addition, glutamate dehydrogenase (GDH), saposin-like domain containing protein, and OsAAA-ATPase are importance in pathogen defense (Pageau et al., 2006; Muñoz et al., 2010; Liu et al., 2020b).

The BPH resistance DEGs were then compared between the different feeding stages. There were 258 DEGs shared only among resistant NILs during either the early or late stages of BPH feeding. Interestingly, there were fewer overlapping DEGs (31 of 258 DEGs) specifically expressed in resistant NILs at the early feeding stage and many more overlapping DEGs (228 of 258 DEGs) specifically expressed in resistant NILs are the late feeding stage. These results imply that certain central signal genes rapidly responded to BPH herbivory at the early stage while many more functional DEGs responded to the signal and were activated to defend against the damage caused by BPH invasion (**Figures 6A, B**). The results of the GO analysis further supported our assumption, as these DEGs were enriched in response to oxidative stress, chloroplast organization, and response to karrikin, all of which are associated with the biotic stress response (**Figure 6C**). Meanwhile, the DEGs were also enriched in secondary metabolite biosynthesis and phenylpropanoid biosynthesis (KEGG) (**Figure 6D**). Ten DEGs were selected as potential BPH resistance candidates, which are associated with either pathogen or herbivore resistance (**Figure 6E**). In rice, the gene *OsATL15* was found to facilitate thiamethoxam accumulation and increase the efficacy of thiamethoxam against BPH (Xiao et al., 2022). In maize, the recessive resistance gene *dissociation inhibitor alpha* (*ZmGDIα*) was found to provide quantitative recessive resistance to maize rough dwarf disease (MRDD) (Liu et al., 2020a). The NAC transcription factors are both negative and positive regulators of downstream defense genes during plant-pathogen interactions. For example, the NAC transcription factor *RIM1* is a negative regulator of rice dwarf virus resistance (Bian et al., 2020). The *lipid transfer protein* (*LTP*) gene coordinates plant resistance to insects and fungi by redirecting metabolic flux (Chen et al., 2021). The peroxidase gene *OsPOX8.1* is strongly induced after pathogen infection, likely through accumulation of ROS (Sun et al., 2014). Here, we confirmed that *OsPOX8.1* could be rapidly and stably induced by BPH infestation, and that overexpression of *OsPOX8.1* in rice protoplasts could increase ROS production (**Figure 1**). Overexpression of *Oryza sativa Rp1-like 1* (*OsRP1L1*) increased resistance to *Xanthomonas* strains *PXO341* and *PXO86* (Wang et al., 2013). In addition, UDP-glucose-dependent glycosyltransferase, late embryogenesis abundant proteins, tubby-like proteins, and cytochrome P450 family proteins are all involved in pathogen defense (Cai et al., 2008; Park et al., 2011; Liu et al., 2013; Wang et al., 2022).

## Conclusion

This was the first endeavor to precisely identify DEGs functionally associated with BPH resistance in NILs pyramiding *BPH14* and *BPH15*. For this purpose, RNA-seq data were generated from 36 mRNA libraries constructed from NILs containing either *BPH14*, *BPH15*, or both *BPH14/BPH15*, as well as their RP, before and after BPH herbivory. The DEGs related to BPH resistance were mainly enriched in defense response and oxidative stress. Additionally, 21 DEGs were chosen as probable BPH resistance candidates by analyzing their expression in different varieties at different feeding stages. One of them, *OsPOX8.1*, was validated in rice protoplasts to increase the accumulation of ROS. Our study not only enhances our understanding of plant-insect interactions in resistance gene pyramiding lines, but will also be foundational for comprehensive functional analyses of the identified candidate DEGs to aid in the improvement of BPH-resistant rice.

## Data availability statement

The datasets presented in this study can be found in online repositories. The names of the repository/repositories and accession number(s) can be found below: https://www.ncbi.nlm.nih.gov/, GSE232449.

## Author contributions

JL, AY, and LH conceived and designed the research. LH, DY, HW, XD, YZ, LN, BW, MX, HQ, and TM participated in the experiments. LH and DY analyzed the data. HW provided the NILs containing *BPH14*, *BPH15*, and *BPH14*/*BPH15*, as well as their recurrent parent. LH, AY, and JL wrote the manuscript. DY and HW helped to edit the manuscript. All authors read and approved the final manuscript.
