# Supplementary material for: Transcriptome analysis revealed differentially expressed genes in rice functionally associated with brown planthopper defense in near isogenic lines pyramiding BPH14 and BPH15
Source: Front Plant Sci. 2023 Aug 8;14:1250590. doi: 10.3389/fpls.2023.1250590 (PMC10442831; doi:10.3389/fpls.2023.1250590)
Supplement: Supplementary file 2 [file Table_2.docx]

**Supplementary Table 2 Summary of mRNA sequence data.**

| Samples | Total reads | Clean reads | Mapped reads | No. of mapped genes |
| --- | --- | --- | --- | --- |
| RP_0-1 | 38,786,156 | 38,362,672  (98.91%) | 34,372,625  (89.60%) | 29,259 |
| RP_0-2 | 40,686,504 | 40,271,930  (98.98%) | 36,044,018  (89.50%) | 29,197 |
| RP_0-3 | 42,906,078 | 42,470,954  (98.99%) | 38,123,580  (89.76%) | 29,336 |
| RP_early-1 | 44,024,448 | 43,516,926  (98.85%) | 38427002  (88.30%) | 29,561 |
| RP_early-2 | 48,056,214 | 47,541,228  (98.93%) | 42,511,732  (89.42%) | 29,608 |
| RP_early-3 | 48,083,072 | 47,553,774  (98.90%) | 42,496,404  (89.36%) | 29,668 |
| RP_late-1 | 46,572,440 | 46,199,270  (99.20%) | 40,947,935  (88.63%) | 29,382 |
| RP_late-2 | 31,739,768 | 31,294,946  (98.60%) | 25,781,469  (82.38%) | 28,869 |
| RP_late-3 | 37,550,164 | 37,025,504  (98.60%) | 32,392,732  (87.49%) | 29,439 |
| B14_0-1 | 46,552,380 | 46,045,630  (98.91%) | 41,418,944  (89.95%) | 29,523 |
| B14_0-2 | 40,488,716 | 40,037,036  (98.88%) | 36,171,079  (90.34%) | 29,238 |
| B14_0-3 | 38,386,272 | 37,971,498  (98.92%) | 34,008,791  (89.56%) | 28,626 |
| B14_early-1 | 47,925,310 | 47,549,816  (99.22%) | 43,047,757  (90.53%) | 29,430 |
| B14_early-2 | 45,507,594 | 44,926,526  (98.72%) | 37,272,639  (82.96%) | 29,221 |
| B14_early-3 | 45,118,074 | 44,634,756  (98.93%) | 39,859,010  (89.30%) | 29,762 |
| B14_late-1 | 37,404,550 | 36,902,102  (98.66%) | 32,708,227  (88.64%) | 29,317 |
| B14_late-2 | 45,675,402 | 45,099,900  (98.74%) | 39,567,717  (87.73%) | 29,836 |
| B14_late-3 | 44,828,022 | 44,303,988  (98.83%) | 38,722,771  (87.40%) | 29,856 |

| Samples | Total reads | Clean reads | Mapped reads | No. of mapped genes |
| --- | --- | --- | --- | --- |
| B15_0-1 | 35,722,718 | 35,232,912  (98.63%) | 31,405,862  (89.14%) | 29,082 |
| B15_0-2 | 43,664,924 | 43,175,916  (98.88%) | 39,063,795  (90.48%) | 29,469 |
| B15_0-3 | 38,848,252 | 38,314,828  (98.63%) | 34,036,640  (88.83%) | 29,181 |
| B15_early-1 | 36,763,650 | 36,275,616  (98.67%) | 31979716  (88.16%) | 29,348 |
| B15_early-2 | 47,083,412 | 46,560,120  (98.89%) | 41,357,463  (88.83%) | 29,498 |
| B15_early-3 | 48,082,854 | 47,440,652  (98.66%) | 40,145,501  (84.62%) | 29,703 |
| B15_late-1 | 43,933,168 | 43,478,614  (98.97%) | 38,103,200  (87.64%) | 29,806 |
| B15_late-2 | 36,328,904 | 35,866,432  (98.73%) | 29,297,676  (81.69%) | 29,210 |
| B15_late-3 | 46,521,614 | 45,962,190  (98.80%) | 40,783,543  (88.73%) | 29,691 |
| B1415_0-1 | 44,765,810 | 44,280,470  (98.92%) | 40,010,018  (90.36%) | 29,430 |
| B1415_0-2 | 37,114,990 | 36,674,106  (98.81%) | 33,097,294  (90.25%) | 29,359 |
| B1415_0-3 | 37,315,162 | 36,944,556  (99.01%) | 33,593,083  (90.93%) | 29,274 |
| B1415_early-1 | 46,718,798 | 46,235,902  (98.97%) | 41,793,871  (90.39%) | 29,937 |
| B1415_early-2 | 50,072,060 | 49,555,078  (98.97%) | 44,922,512  (90.65%) | 29,831 |
| B1415_early-3 | 39,619,100 | 39,090,472  (98.67%) | 34,551,235  (88.39%) | 29,530 |
| B1415_late-1 | 43,405,304 | 42,954,354  (98.96%) | 37,725,930  (87.83%) | 29,691 |
| B1415_late-2 | 48,865,688 | 48,302,064  (98.85%) | 43,205,877  (89.45%) | 29,860 |
| B1415_late-3 | 45,876,230 | 45,352,418  (98.86%) | 40,451,504  (89.19%) | 29,704 |

Note:

Total Reads: the raw data after sequencing. Clean Reads: the reads after filtering out low-quality tags, unexpected-length tags, and single-copy tags. Mapped Reads: the reads of the clean reads that could be mapped to the reference genome.

RP: recurrent parent ‘Wushansimiao’ for NILs; B14, B15, and B1415: the NILs containing the *BPH14*, *BPH15*, and both *BPH14/BPH15* genes, respectively. 0, non-infested; early: early feeding stage; late: late feeding stage.
